# A novel phytopathogen *Erwinia sorbitola* sp. nov., isolated from the feces of ruddy shelducks

**DOI:** 10.3389/fcimb.2023.1109634

**Published:** 2023-02-16

**Authors:** Yuanmeihui Tao, Yajun Ge, Jing Yang, Weitao Song, Dong Jin, Hong Lin, Han Zheng, Shan Lu, Wenbo Luo, Yuyuan Huang, Zhenhong Zhuang, Jianguo Xu

**Affiliations:** ^1^ State Key Laboratory of Infectious Disease Prevention and Control, National Institute for Communicable Disease Control and Prevention, Chinese Center for Disease Control and Prevention, Changping, Beijing, China; ^2^ College of Basic Medicine, Gansu University of Chinese Medicine, Lanzhou, China; ^3^ Research Units of Discovery of Unknown Bacteria and Function, Chinese Academy of Medical Sciences, Beijing, China; ^4^ Key Laboratory of Pathogenic Fungi and Mycotoxins of Fujian Province, Proteomic Research Center, and School of Life Sciences, Fujian Agriculture and Forestry University, Fuzhou, China; ^5^ Research Institute of Public Health, Nankai University, Tianjin, China

**Keywords:** phytopathogen, reverse microbial etiology, pathogenicity analysis, *Erwinia sorbitola*, ruddy shelducks

## Abstract

The species in the genus Erwinia are Gram-stain-negative, facultatively anaerobic, motile, and rod-shaped. Most species in the genus Erwinia are phytopathogens. Also, Erwinia persicina was involved in several human infections. Based on the reverse microbial etiology principles, it is worth analyzing the pathogenicity of species in this genus. In this study, we isolated and sequenced two species of Erwinia. Phylogenetic, phenotypic, biochemical, and chemotaxonomic analyses were performed to identify its taxonomy position. The virulence tests on plant leaves and pear fruits were used to identify the plant pathogenicity of two species of Erwinia. Bioinformatic methods predicted the possible pathogenic determinants based on the genome sequence. Meanwhile, adhesion, invasion, and cytotoxicity assays on RAW 264.7 cells were applied to identify animal pathogenicity. We isolated two Gram-stain-negative, facultatively anaerobic, motile, and rod-shaped strains from the feces of ruddy shelducks in the Tibet Plateau of China, designated J780T and J316. Distinct phylogenetic, genomic, phenotypic, biochemical, and chemotaxonomic characters of J780T and J316 identified they were novel species and belonged to the genus Erwinia, for which the name Erwinia sorbitola sp. nov. was proposed, the type strain was J780T (= CGMCC 1.17334T = GDMCC 1.1666T = JCM 33839T). Virulence tests showed blight and rot on the leaves and pear fruits confirmed Erwinia sorbitola sp. nov. was a phytopathogen. Predicted gene clusters of motility, biofilm formation, exopolysaccharides, stress survival, siderophores, and Type VI secretion system might be the causes of pathogenicity. In addition, predicted polysaccharide biosynthesis gene clusters on the genome sequence, and the high capacity for adhesion, invasion, and cytotoxicity to animal cells confirmed it has pathogenicity on animals. In conclusion, we isolated and identified a novel phytopathogen Erwinia sorbitola sp. nov. in ruddy shelducks. A predefined pathogen is beneficial for preventing from suffering potential economic losses caused by this new pathogen.

## Introduction

The genus *Erwinia* was first described by Winslow et al., with *Erwinia amylovora* being the type species. Cells of the genus *Erwinia* are mainly characterized as Gram-stain-negative, facultatively anaerobic, motile, rod-shaped bacteria with 49.8-54.1 mol% of G+C contents (Hauben et al., 1998). Their major fatty acids are C_12:0_, C_14:0_, and C_16:0_ (Hauben et al., 1998). Thus far, there are 20 species in the genus *Erwinia* with valid names (https://lpsn.dsmz.de/genus/erwinia). Most strains are plant pathogens, including *E. amylovora* (Hauben et al., 1998), *E. aphidicola* (Harada et al., 1997), *E. mallotivora* (Amin et al., 2010), *E. papaya* (Gardan et al., 2004), *E. pyrifoliae* (Kim et al., 1999), *E. piriflorinigrans* (Lopez et al., 2011), and *E. uzenensis* (Matsuura et al., 2012). Among these phytopathogens, *E. amylovora* can cause fire blight, a plant disease which threatens host species, including *Maloideae* (commercial apple and pear), *Rosoideae* (blackberry and raspberry), and *Spiraeoideae*. The pathogenesis of *E. amylovora* is that cells enter susceptible hosts through the nectarthodes of flowers and other natural openings, rapidly moving through the parenchyma and then establishing a systemic infection through the xylem vessels, finally provoking extensive lesions; in late stages, the infected plants will appear brown or black (Pique et al., 2015).

In the pathogenic mechanism of the genus *Erwinia*, the Type III secretion system (T3SS), the exopolysaccharide (EPS) amylovoran, biofilm formation, and motility were characterized as virulent determinants in species of *Erwinia* (Kharadi et al., 2021). Due to its pathogenicity to many host plants (including many fruit plants), it is necessary to conduct in-depth research into it.

Subsequent studies have also found that: *E. persicina* has been isolated from human urine (O'Hara et al., 1998); *E. tasmaniensis* was involved in a cervical lymphadenitis case (Shin et al., 2008b); and *E. billingiae* has been associated with dermohypodermitis (Prod'homme et al., 2017) and septic arthritis (Bonnet et al., 2019).

The *Erwinia sorbitola* sp. nov. was isolated from ruddy shelduck (*Tadorna ferruginea*), a migratory species that were involved in the highly pathogenic avian influenza (HPAI) H5N1 that broke out at Qinghai Lake (Liu et al., 2005). Due to migrating large distances between their breeding and wintering areas, several novel microorganisms have been isolated from ruddy shelduck (Marinova et al., 2015; Ge et al., 2020), which is a reminder it may be a potential vector of more novel pathogens.

Advanced pathogen isolation and identification are efficient ways to avoid spreading and properly treat infectious diseases. There have been several successful reverse microbial etiology cases reported recently. *Anaplasma capra* was first isolated and identified, and then the patients related to this *Anaplasma* were informed (Li et al., 2015b; Wei et al., 2017). Additionally, *Escherichia marmotae* was identified as a human pathogen when four infected cases were reported to have been caused by *Escherichia marmotae* after our team first isolated it in 2015 (Liu et al., 2015; Liu et al., 2019; Sivertsen et al., 2022).

Herein, we characterized a novel species in the genus *Erwinia* (named *Erwinia sorbitola* sp. nov.) from the potential microbial pathogen vector ruddy shelduck, and the pathogenicity of this pathogen on plants and animals was explored in this study.

## Materials and methods

### Isolation and cultural conditions

Immediately after ruddy shelducks (*T. ferruginea*) took flight, droppings samples (20 samples) of these birds were freshly collected from the ground and stored in sterile tubes. The fecal samples were collected at two different geographical locations (E 91°48′22″/N27°54′23″ and E 91°58′53″/N27°59′16″) in Cona County, Tibet. Pieces of material (approximate 1-2 g) were diluted in sterile water from 10^-1^ to 10^-6^ and homogenized by shaking, then 100 μL of the 10^-4^ dilution was spread onto Nutrient Agar (NA) plates. After five days of aerobic incubation at 28 °C, different morphological colonies were randomly picked and separated. Strains J780^T^ and J316 from among all the beige and opaque colony isolates (J178, J312, J316, J556, and J780^T^) were selected based on a comparative analysis of 16S rRNA gene sequences. The reference strains *E. aphidicola* DSM 19347^T^, *E. persicina* GDMCC 1.331^T^, and *E. rhapontici* ATCC 29283^T^ were obtained from the Japan Collection Microorganisms (JCM), Guangdong Microbial Culture Collection Center (GDMCC), and China General Microbiological Culture Collection Center (CGMCC), respectively.

### Phylogenetic analyses of strains J780^T^ and J316

The genomic DNA of strains J780^T^ and J316 was extracted using the Wizard Genomic DNA Purification Kit (Promega). The 16S rRNA genes were cloned by universal bacterial primers 27F/1492R (5’-agagtttgatcmtggctcag-3’and 5’-ggytaccttgttacgactt-3’) (Ge et al., 2020) and deposited into the GenBank nucleotide database of NCBI (length 1,507 bp, accession numbers MN203624 and MN708966). The sequence similarities to the related type strains were calculated using BLASTn. Sequences of novel and related type strains were aligned using the Clustal_W program (Larkin et al., 2007). Phylogenetic trees based on 16S rRNA gene sequences were constructed with the software package MEGA 7.0 (Kumar et al., 2016), through the algorithms of neighbor-joining (NJ) (Saitou and Nei, 1987), maximum-likelihood (ML) (Felsenstein, 1981), and maximum parsimony (MP) (Kolaczkowski and Thornton, 2004). Confidence levels of branching were determined by bootstrap analysis with 1,000 replicates (Felsenstein, 1985). *Pseudomonas aeruginosa* LMG 1242^T^ was used as an outgroup.

### Analysis of the morphological, physiological, and biochemical characterization of strains J780^T^ and J316

The optimal growth conditions for strains J780^T^ and J316 were detected in nutrient broth at different temperatures (4, 16, 28, 30, 35, 37, and 42°C), salinities [0.5–10% (w/v) NaCl at intervals of 0.5%], and pH [(3–12) at intervals of 1.0 pH unit]. Cell morphology, Gram-staining, and colony appearance were observed using isolated bacterial stains grown on NA plates at 28°C for 24 h. Cell morphology was observed by a scanning electron microscope (SU8010, Hitachi). Gram-staining was performed using a Gram-staining kit (Baso) (Austrian, 1960). With *E. aphidicola* DSM 19347^T^, *E. persicina* GDMCC 1.331^T^, and *E. rhapontici* ATCC 29283^T^ as positive controls for the motility assay, cell motility was tested by growing in semi-solid agar at 22°C for 48 h (Xu et al., 2013). The catalytic activity of catalase was detected using 3% (v/v) H_2_O_2_. Enzymatic activity and acid production from various carbohydrates of the novel strains were determined by API ZYM, API 50 CHB, API 20E, and API 20NE strips (bioMérieux), and strains *E. aphidicola* DSM 19347^T^, *E. persicina* GDMCC1.331^T^, and *E. rhapontici* ATCC 29283^T^ were used as the parallel references.

### Chemotaxonomic characterization

Chemotaxonomic analyses were performed after bacteria had grown on NA plates at 28°C for 24 h. The cellular fatty acids were extracted and analyzed in parallel according to the instructions of the Sherlock Microbial Identification System (MIDI) (Sasser, 1990). The respiratory quinones were analyzed by HPLC (Minnikin et al., 1984; Tamaoka, 1986).

### General genomic analysis of strains J780^T^ and J316

The whole genome sequence of strain J780^T^ was determined using PacBio RSII single-molecule real-time (SMRT) sequencing (PacBio). Through the hierarchical genome assembly process (HGAP) protocol (Version 4.0) in SMRT Analysis (Version 2.3.0), *de novo* assembly of sequencing reads was performed, and one circular genome without gaps was generated. With strains *E. aphidicola* DSM 19347^T^ and *E. rhapontici* ATCC 29283^T^ as references, strain J316 was sequenced on the Illumina X Ten platform following a paired-end protocol and assembled by the SOAP *de novo* (Illumina) (Luo et al., 2012; Li et al., 2015a), and the protein-coding sequences (CDSs), tRNAs, and rRNAs were predicted using GeneMarkS+4.2 software (Berlin et al., 2015). The average nucleotide identity (ANI) and digital DNA-DNA hybridization (dDDH) values were examined with the OrthoANIu tool (https://www.ezbiocloud.net/tools/ani) (Yoon et al., 2017) and the genome-to-genome distance calculation (GGDC 2.1) (Auch et al., 2010; Meier-Kolthoff et al., 2013), respectively. To further elucidate the taxonomy position of strains J780^T^ and J316 in the phylogenetic relationship, core genes (Chen et al., 2013) were extracted from the genome sequences of the isolates and 13 species in the genus *Erwinia* and aligned to generate the phylogenomic tree through FastTree (Price et al., 2009). The phylogenetic tree was further edited with Dendroscope.

### Prediction of pathogenicity

The pathogenicity of isolates was predicted by the Pathogen-Host Interactions database (PHI) and the Virulence Factors of Pathogenic Bacteria (VFDB). Amino acid sequences were compared in the databases by the program DIAMOND (cutoff: 40% identity). When the annotation results were over one, the best annotation was selected by ensuring the biological functions. The arrangement and similarities of gene clusters were illustrated with Easyfig (Version 2.2.2). The amino acid similarities of type VI secretion system effector genes were compared through BlastP on the NCBI website (https://blast.ncbi.nlm.nih.gov/Blast.cgi).

### Virulence assays on plants

The virulence tests on these bacterial isolates were performed by adopting leaf-clipping and pear-fruit inoculation methods as previously described (Dow et al., 2003; Tian et al., 2017). The leaf models included arabidopsis (*Arabidopsis thaliana*, At), tobacco (*Nicotiana benthamiana*, Nb), potato (*Solanum tuberosum*, St), Chinese flowering crabapple (*Malus halliana*, Mh), and cherry blossom (*Prunus campanulate*, Pc), and the plant leaves were gifted by FAFU, (Fujian Agriculture and Forestry University). The pear fruits were purchased from a commercial market (Changping, Beijing). In short, the bacteria (J178, J312, J316, J556, J780^T^ *, E. aphidicola* DSM 19347^T^, and *E. persicina* GDMCC 1.331^T^) were harvested at the logarithmic phase by centrifugation, and the bacterial precipitates were washed with PBS three times before inoculating the plants. The leaves were clipped and inoculated with an equal load of bacteria. Pear fruits were inoculated with 100 μL bacteria suspension through disposable needles (1 mL range). All the infection models were incubated in high humidity at 28°C. Symptoms were observed and recorded within the following days.

### Bacterial adhesion, invasion, and cytotoxicity assays

Adhesion assay was done according to the following description (Chen et al., 2017). RAW264.7 cells (mice macrophage) were cultured in DMEM (supplemented with 10% FBS), and 1×10^5^ cells/well were plated to 24 multi-well plates one day before the experiment. 1×10^7^ CFU/well logarithmic phase bacteria (strain: J178, J312, J316, J556, and J780^T^) were mixed with RAW264.7 cells washed with PBS after they were centrifuged and resuspended in DMEM (without FBS). Plates were incubated at 37°C supplied with 5% CO_2_ for two hours, then washed five times with PBS. The infected RAW264.7 cells were lysed for 10 min with 1% saponin (Sigma-Aldrich), and the lysates were diluted with PBS from 1:1000 to 1:10000.

Based on the above, the invasion assay was conducted through another two hours of antibiotic treatments. The infected RAW264.7 cells were also lysed for 10 min with 1% saponin (Sigma-Aldrich), and the lysates were diluted with PBS from 1:100 to 1:1000. Plate count was adopted for quantitation of the colony forming unit (CFU) of live bacteria in samples. All the assays were done three times. The cytotoxicity assay was carried out following the protocol of the CytoTox 96^®^ Non-Radioactive Cytotoxicity Assay (Promega).

### Accession numbers

The GenBank/EMBL/DDBJ accession numbers for the 16S rRNA gene sequence of strains J780^T^ and J316 are MN203624 and MN708966, respectively. The GenBank/EMBL/DDBJ accession numbers for the genome sequence of strains J780^T^, J316, *E. rhapontici* ATCC 29283^T^, and *E. aphidicola* DSM 19347^T^ are CP046509–CP046510, WLZX00000000, WUUO00000000, and JACXBP000000000, respectively.

## Results

### Physiological, phenotypic, biochemical, and chemotaxonomic characteristics

The bacterial cells of J780^T^ grown on the NA plate were observed under the scanning electron microscope. The result showed that the J780^T^ strain was rod-shaped (1.5-2.0 mm in length and 0.6-1.0 mm in diameter and Gram-stain-negative (stained with a Gram-staining kit (Baso) (Austrian, 1960)). The catalase activity was tested by treating the J780^T^ strain with 3% (v/v) H_2_O_2_, and the results showed that the enzymatic activity of this strain was positive. By growing in semi-solid agar at 22°C for 48 h, the observed results showed that the J780^T^ strain was motile. The colonies of the strain J780^T^ grown on NA were circular, low convex, smooth, entire edge, and beige. It was different from the pink color of *E. persicina* GDMCC 1.331^T^ grown on TSA at 28°C for 24 h. Through the research on serial growth conditions, it was found that the best combination conditions for growth in both strains J780^T^ and J316 were under 28°C (the screening range: 4-40°C), at pH7.0 (pH4.0-10.0), and with 0.5% (w/v) NaCl (0.5-5.0% (w/v)).

All strains (including J780T, J316, *E. persicina*, and *E. rhapontic*) emerged in a state of diffusion growth within 24 h when inoculated into semi-solid agar at 22°C. Any one of these tests, including the tests for N-acetyl-*β*-glucosaminidase, potassium 5-ketogluconate, and citrate, d-Sorbitol, and d-Turanose utilization, could distinguish the strains J780^T^ and J316 from their closely related species *E. aphidicola* DSM 19347^T^, *E. persicina* GDMCC 1.331^T^, and *E. rhapontici* ATCC 29283^T^ (**Table 1**).

**Table 1:** Phenotypic characteristics, cellular fatty acid composition (percentages), and respiratory quinone differentiate *Erwinia sorbitola* sp. nov. and other species from *Erwinia*.

	Items	J780T	J316	E. aphidicola	E. persicina	E. rhapontici
**Morphological, physiological characterization**	Colony color	beige	beige	ivory	pink	light beige
	NaCl(w/v) range(optimum)	0.5–5 (0.5)	0.5–5 (0.5)	0.5–5 (0.5)	0.5–5 (0.5)	0.5–5 (0.5)
	pH range (optimum)	4–10 (7)	4–10 (7)	4–10 (7)	4–10 (7)	4–10 (7)
	Temperature (℃) (optimum)	4–40 (28)	4–40 (28)	4–37 (28)	4–37 (28)	4–40 (28)
**Enzyme activity**	N-acetyl-*b*-glucosaminidase	+	+	–	–	–
	*a*-glucosidase	–	–	+	–	+
	ornithine decarboxylase	+	+	+	–	–
	catalase	+	+	+	+	+
	citrate utilization	–	–	+	+	+
**Acid formation (API 50CH)**	d-Lactose	–	–	–	+	+
	Potassium gluconate	–	+	–	–	–
	Potassium 5-ketogluconate	+	+	–	–	–
	d-Sorbitol	+	+	–	+	+
	d-Turanose	–	–	–	–	+
**Fatty acid (%)**	C_16:0_	**24.8**	**26.7**	**30**	**26.8**	**28.4**
	C_17:0 cyclo_	7.5	9.5	**10.1**	**18.6**	**13.5**
	Summed feature 3^*^	**16.3**	**28.7**	**22.6**	**20.3**	**23.3**
	Summed feature 8*	**7.2**	**11.7**	**10.9**	**8.9**	**11.5**
**Respiratory quinone**	Major respiratory quinone	Q9/Q8/Q10	NA	NA	NA	NA

﻿TABLE 1 Phenotypic characteristics, cellular fatty acid composition (percentages), and respiratory quinone differentiate Erwinia sorbitola sp. nov. and other species from Erwinia.

﻿All data was taken from this study unless otherwise indicated. +, Positive; -, Negative; NA, not available.

* Summed features are fatty acids that cannot be resolved reliably from another fatty acid using the chromatographic conditions chosen. The MIDI system groups these fatty acids together as one feature with a single percentage of the total. Summed feature 3 containing C16:1w7c and/or C16:1w6c; Summed feature 8 containing C18:1w7c and/or C18:1 w6c.

The major cellular fatty acids were determined by Sherlock Microbial Identification System (MIDI) (Sasser, 1990). The results showed that the major cellular fatty acids (>10%) in strain J780^T^ and J316 were C_16:0_ (24.8%, 26.7%), summed feature 3 (C_16:1_ *ω*7c_/16:1_ *ω*6c, 16.3%, 28.7%), summed feature 2 (C_14:0_ 3OH and/or C_16:1_ iso I, 15.8%, 7.1%), and summed feature 8 (C_18:1ω7c_ and/or C_18:1 ω6c_ 7.2%, 11.7%). The major respiratory quinones of strain J780^T^ were Q9 (78.5%) with minor Q8 (17.9%) and Q10 (3.6%) (**Table 1**), which was different from the control strain *E. endophytica* LMG 28457^T^, the only known species in the genus *Erwinia* with only determined respiratory quinone Q8 (Ramirez-Bahena et al., 2016).

### Phylogenetic analysis

**Figure 1:**
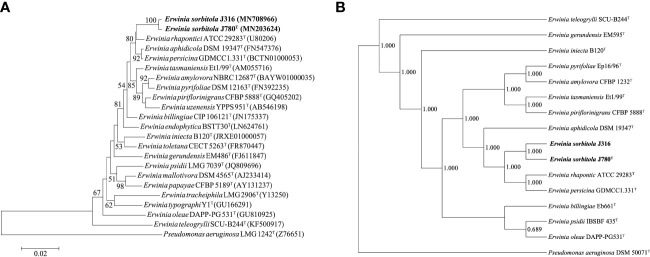
The phylogenetic analyses of *Erwinia sorbitola* sp. nov. and other species from *Erwinia.* **(A)** Neighbor-joining phylogenetic tree based on partial 16S rRNA gene sequences, showing the relationships between strains J316, J780^T^, and members of the genus *Erwini*a (Bootstrap values (> 50%) calculated for 1,000 replications are shown at branch nodes; Bar, 0.02 substitutions per nucleotide); **(B)** the Phylogenomic tree of strains J316 and J780^T^ based on 916 core genes (numbers on the core gene tree indicate each split in the tree with support values from the Shimodaira-Hasegawa test calculated for 1,000 resamples). *Pseudomonas aeruginosa* LMG 1242^T^ was used as the outgroup.

The sequencing and comparison results showed that the sequence of the 16S rRNA gene of strain J780^T^ shared 98.2% similarity with that of *E. rhapontici*, 98.1% with *E. persicina*, and 97.9% with *E. piriflorinigrans* CFBP 5888^T^. In contrast, strain J316 displayed 98.3%, 98.0%, and 97.8% similarity with these strains, respectively. Strains J780^T^ and J316 shared up to 99.7% 16S rRNA gene sequence similarity while sharing less than 98.7% 16S rRNA gene sequence similarity to any other strains in the genus *Erwinia*. In the phylogenetic tree constructed based on 16S rRNA genes (**Figure 1A**; **Supplementary Figure 1**), strains J780^T^ and J316 clustered with *E. rhapontici* ATCC 29283^T^, *E. aphidicola* DSM 19347^T^, and *E. persicina* GDMCC1.331^T^ were phylogenetically closest to *E. rhapontici* ATCC 29283^T^. Besides 16S rRNA gene-based analysis, the phylogenetic tree based on 916 core gene sequences (**Figure 1B**) also showed similar results of classification that strains J780^T^ and J316 were closest with *E. rhapontici* ATCC 29283^T^ and *E. persicina* GDMCC1.331^T^, which supporting the affiliation of strains J780^T^ and J316 to the genus *Erwinia*.

The ANI values of strains J780^T^ and J316 with the closely related species *E. aphidicola* DSM 19347^T^ *, E. persicina* GDMCC1.331^T^, and *E. rhapontici* ATCC 29283^T^ ranged from 81.7 to 85.0%, while the dDDH values ranged from 25.1 to 29.5%. The ANI and dDDH values between strains J780^T^ and J316 were 99.6 and 96.5%, respectively. Furthermore, the values of ANI and dDDH between each isolate and *Erwinia* species were lower than the proposed threshold of 95-96 and 70%, respectively, upholding the view that strains J780^T^ and J316 were novel species of the genus *Erwinia*.

In conclusion, multiple in-depth analyses based on phylogenetic, physiological, and chemotaxonomic properties indicated that strains J780^T^ and J316 belonged to the genus *Erwinia*. Combined with the ANI and dDDH values compared with other species in the genus, as well as differential phenotypic properties and distinguishing fatty acid profiles, this supported the proposal that these two isolates (strains J780^T^ and J316) were a novel species, for it could utilize d-Sorbitol, which was different from its reference strains, the study named it *Erwinia sorbitola* sp. nov.

### Pathogenicity on plants

Most of the species in the genus *Erwinia* have always been known as plant pathogens. Therefore, we compared the genomes of both isolates to the PHI database using the program DIAMOND for screening the plant disease-related genes. The search showed that a few cluster genes might cause plant blight diseases (host: pear and apple) and black rot (host: radish). The detailed information is shown in **Supplementary Table 1**.

**Figure 2:**
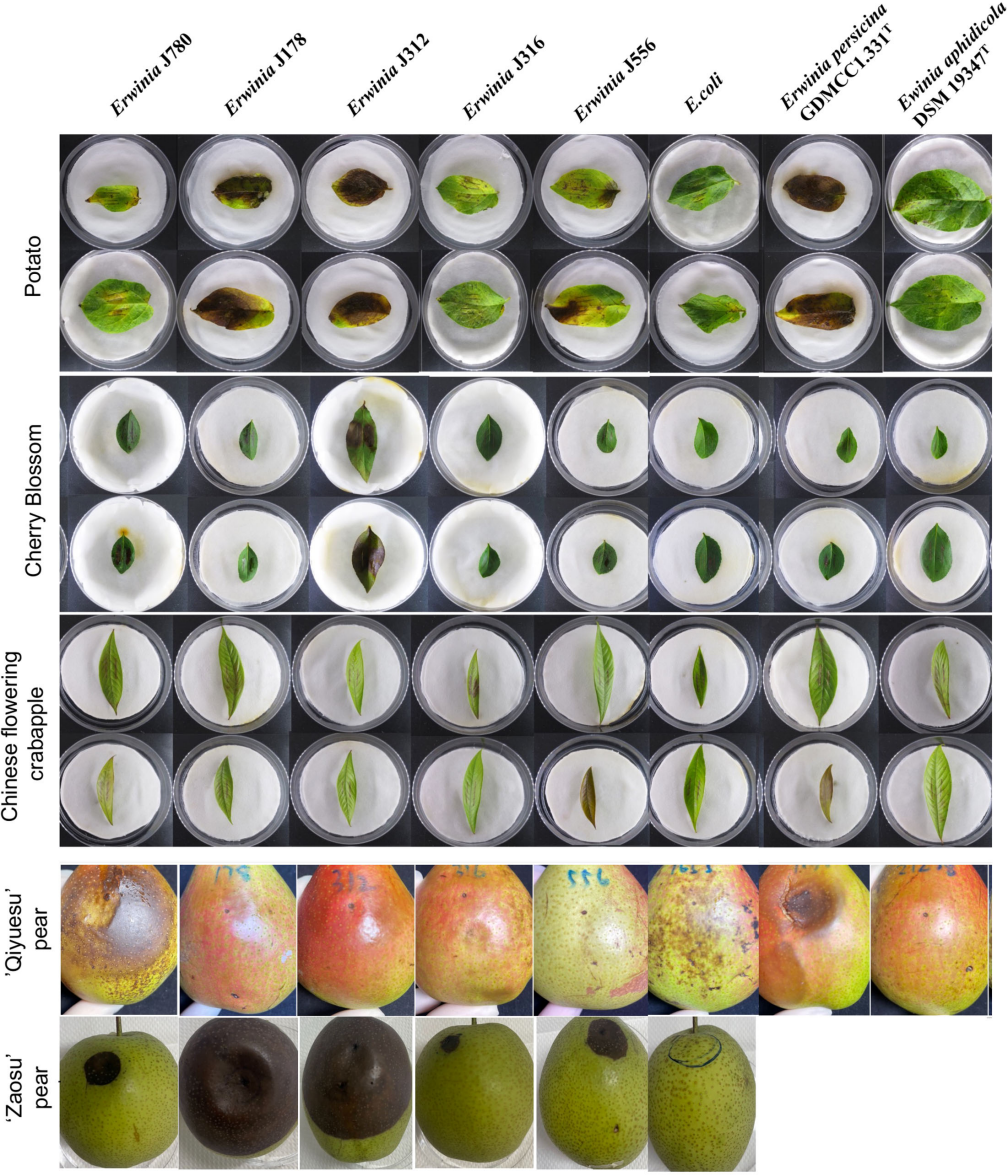
The virulence tests on leaves and pear fruits of *E. sorbitola* sp. nov. and the type strains of the reference strains (strains: J780^T^, J178, J312, J316, and J556; reference strains: *E. persicina* GDMCC 1.331^T^ and *E. aphidicola* DSM 19347^T^). The virulence tests on leaves of *E. sorbitola* sp. nov. were conducted *via* leaf-clipping method (leaves: potato, cherry blossom, Chinese flowering crabapple); the virulence tests on fruits of *E. sorbitola* sp. nov. were conducted *via* injection into pear fruits by needle (fruits: ‘Qiyuesu’ pear, ‘Zaosu’pear). The infection dose was 1×10^7^ CFU/mL, leaves were incubated for three days, and fruits were incubated for 14 days. *E. coli* was used as negative control strain. Experiments were repeated twice.

According to the results of the PHI database analysis, the leaves of the three plants, including Arabidopsis (*A. thaliana*, At), tobacco (*N. benthamiana*, Nb), and potato (*S. tuberosum* L., St), and the fruits of two *Rosaceae* plants, including Chinese flowering crabapple (*M. halliana*, Mh) and cherry blossom (*P. campanulate*, Pc) were constructed as infection models in this study. The result displayed no obvious infection symptoms on the leaves of both arabidopsis and tobacco compared to that of the control group, *E. coli*. However, all the strains (J780^T^, J178, J312, J316, J556, *E. aphidicola* DSM 19347^T^, and *E. persicina* GDMCC1.331^T^) caused different levels of the symptoms like the burnt by the fire on potato and cherry blossom leaves, in which strains *E. persicina* GDMCC1.331^T^, J178, and J312 induced more severe symptoms on the leaves of potato than the other strains. Moreover, a slightly burnt symptom was found on the leaves of Chinese flowering crabapple in all the experimental groups (**Figure 2**). All the experiments were repeated twice.

In the fruit models, pears fruits were inoculated with isolates, and *E. coli* was taken as the negative control. Depending on the different bacterial species infected, a discrepancy in the appearance of infected symptoms was present on the infected pear fruits. After 14 d inoculation, the pear fruits (‘Qiyuesu’ pear (*P. pseudopashia*, Pp), ‘Zaosu’ pear (*P. bretschneideri* cv. *Zaosu*, Pbz), and ‘Anhui Dangshan’ pear (*P. bretschneideri Rend*, Pbr)) infected by J780^T^ and *E. persicina* GDMCC1.331^T^ showed symptoms of rot, but no obvious appearance of infected symptom showed up on the pears infected by the other bacterial strains (**Figure 2**, ‘Qiyuesu’ pear). After 14 d inoculation, the pear fruits (‘Zaosu’ pear) inoculated with strains of *E. sorbitola* sp. nov. all presented symptoms of water-soaking and necrosis-infection, but no obvious infected symptoms were developed in the control group (**Figure 2**, ‘Zaosu’ pear). A similar test was carried out with the ‘Anhui Dangshan’ pear (*Pyrus bretschneideri*, Pb) and ‘Guoguang’ apple (*Ralls Janet*, Rj) fruits. The results showed that symptoms of rot were present on the pears infected by *Erwinia persicina* GDMCC1.331^T^ (**Supplementary Figure 2**), but no symptom could be observed in the apple model (data not shown). These results proposed that, similar to other members of the genus *Erwinia*, the novel isolates of *E. sorbitola* sp. nov. were phytopathogenic to pear fruits, and *E. persicina* GDMCC1.331^T^ may be a hypervirulent strain with a wide range of susceptible hosts.

### Pathogenesis analysis on plants based on virulence factors

**Figure 3:**
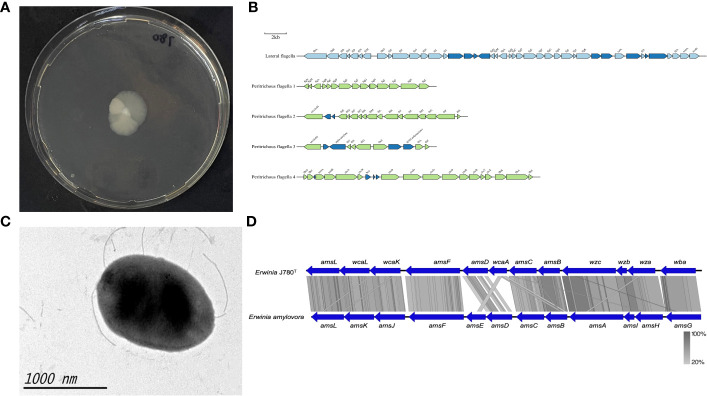
The motility and amylovoran biosynthesis genes of *E. sorbitola* sp. nov. (strain: J780^T^). **(A)** Bacterial swimming assay of the strain J780^T^; **(B)** schematic representation of flagella gene clusters of the strain J780^T^. Proposed gene nomenclature is shown above the corresponding ORFs (the scale bar represents 2kb); **(C)** transmission electron micrograph of the strain J780^T^ (the scale bar represents 1000nm); **(D)** the comparison of amylovoran biosynthesis genes between the strain J780^T^ and *Erwinia amylovora.* The figure was compared using BlastX and pictured with Easyfig.

Considering the phytopathogenic phenotype of these two isolates of *Erwinia*, their potential virulence factors were characterized by analysis through the VFDB and PHI database based on the genomes of both isolates. Moreover, the results showed that the pathogenicity of this novel bacterium might be determined by the following pathogenic characteristics, including motility (**Supplementary Table 2**), biofilm formation (**Supplementary Table 3**), and exopolysaccharides (EPS) amylovoran **Figure 3D**; stress survival abilities and siderophores (**Supplementary Table 5**); and Type VI secretion system (**Supplementary Table 6**). Based on the predicted results, serial follow-up experiments were carried out.

### Motility, biofilm formation, and exopolysaccharides amylovoran

The motility of J780^T^ was tested *via* bacterial swimming assay. The result showed that it possessed high motility (**Figure 3A**). Prediction information from this study inferred that it might own lateral flagella and peritrichous flagella (**Figure 3B**). The detailed flagella related encoding genes are listed in a supplementary table (**Supplementary Table 2**). Moreover, other visualization under transmission electron microscopy confirmed that its flagella played an essential role in its high motility (**Figure 3C**). Meanwhile, our prediction information also revealed that there were four gene clusters related to pili, including type I fimbriae, PMF fimbriae, mannose-resistant fimbriae, and separated genes on its genome with the type IV pili annotation (**Supplementary Table 3**). The amylovoran synthesis gene cluster contains 12 structural genes (from *amsA* to *amsL*) (Bugert and Geider, 1995) in the chromosome of *E. amylovora*. In this study, a similar *ams* gene cluster associated with amylovoran biosynthesis was screened from the genomes of J780^T^ (**Figure 3D**), and related detailed information is given in the supplementary materials (**Supplementary Table 4**). The above results displayed that the characteristics, including motility, biofilm formation, and exopolysaccharides (EPS) amylovoran, were deeply involved in the formation of virulence of this novel bacterium to host plants.

### Stress survival and Siderophores of *E. sorbitola* sp. nov.

**Figure 4:**
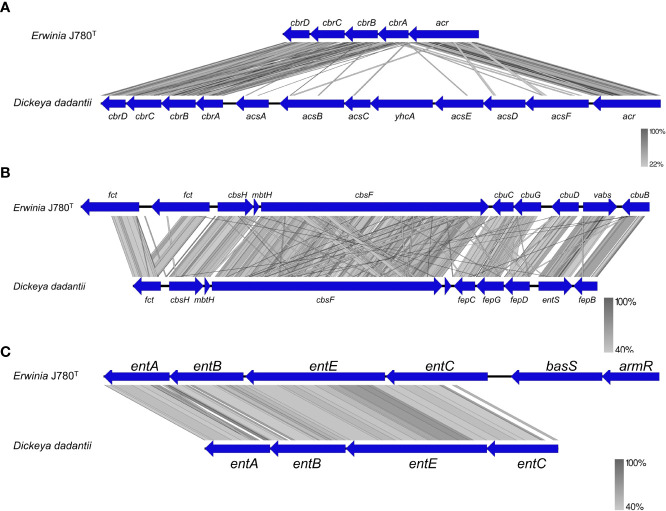
The comparison of siderophores genes between *E. sorbitola* sp. nov. (J780T) and *Dickeya dadantil.* **(A)** The comparison of achromobactin gene clusters; **(B)** the comparison of chrysobactin gene clusters; **(C)** the comparison of enterobactin gene clusters. The proposed gene nomenclature is shown above the corresponding ORFs. All the figures were compared using BlastX and pictured with Easyfig.

Resistance and adaptation to hostile environments are essential virulence factors for pathogens. According to VFDB prediction in this study, genes associated with the pathogens’ stress survival, including catalase and metal ion transport, were screened from the genome of *E. sorbitola* sp. nov. (**Supplementary Table 5**). In these predicted associated genes, at least three genes, including *katA* (J780_GM000409), *katG* (J780_GM000646), and *ahpC* (J780_GM001015), were coding as peroxiredoxin related with catalase activity, which was consistent with the positive results of catalase activity in this study. The iron uptake systems, including the direct heme uptake system (Hogle et al., 2017), ferrous iron (Fe^2+^) transport (Seo et al., 2014), and siderophores (Aznar et al., 2014), played an indispensable role in bacteria survival in iron-limited conditions and regulation of virulence activities. There were three siderophores predicted in the genomes of the novel isolates in this study. They were siderophores chrysobactin (Rauscher et al., 2002; Sandy and Butler, 2011), achromobactin (Berti and Thomas, 2009), and enterobactin (Gorshkov et al., 2021). Notably, in this study, the three predicted siderophores in *E. sorbitola* sp. nov. showed high similarities to those of *Dickeya dadantii* (**Figures 4A–C**). These results suggested that catalase and metal ion transport play an essential role in the pathogenic process of *E. sorbitola* sp. nov. to its host plants.

### Type VI secretion system of *E. sorbitola* sp. nov.

There were four gene clusters related to the Type VI secretion system (T6SS), including three integrated T6SSs and one auxiliary gene cluster. The T6SS constitutions of J780^T^ are shown in **Supplementary Table 6**. The auxiliary gene cluster started at the locus J780_GM000434 and ended with J780_GM000441, which contained 8 ORFs. Among these ORFs, the PIN may be one of the effector candidates of the auxiliary gene cluster. The other integrated T6SSs had 21, 24, and 28 ORFs containing all 13 ORFs core genes of the T6SS (Chen et al., 2015; Crisan and Hammer, 2020). T6SS-1 was from J780_GM001628 to J780_GM001644, and T6SS-2 clusters were from J780_GM002048 to J780_GM002071. The T6SS cluster 3 (from J780_GM003723 to J780_GM003746) structure was like *Citrobacter rodenthum* CTS1(T6SS1) but with an additional fimbriae operon in it (Gueguen and Cascales, 2013).

The T6SS-2 and auxiliary gene clusters in the J780^T^ genome shared homologs with *E. amylovora*, as predicted by the PHI database, are listed in **Supplementary Table 7**. Almost all the T6SS core components of *E. sorbitola* sp. nov. shared homologous with *E. amylovora*, and it was reported that the deletion of T6SS structure components and effectors in *E. amylovora* caused a decrease in amylovoran production and reduction in bacteria virulence (Tian et al., 2017). These findings suggested that T6SS-2 and auxiliary clusters may function as phytopathogenic determinants in *E. sorbitola* sp. nov. as well.

### Preliminary analysis on animal pathogenicity of *E. sorbitola* sp. nov.

This study predicted several polysaccharide biosynthesis gene clusters, including LOS, LPS, and CPS. They were involved in immune system evasion and anti-phagocytosis (**Supplementary Table 4**). Type I fimbriae and mannose-resistant fimbriae also functioned as adhesive factors (**Supplementary Table 3**). They may promote the adherence of pathogens to bladder cells and macrophages (Mohammad Zadeh et al., 2021). These predictions inferred that *E. sorbitola* sp. nov. may have high adhesion and invasion abilities. To identify the adhesion, invasion abilities, and cytotoxicity of the bacteria, we counted the bacteria burdens attached to the cell surfaces and invaded cells.

**Figure 5:**
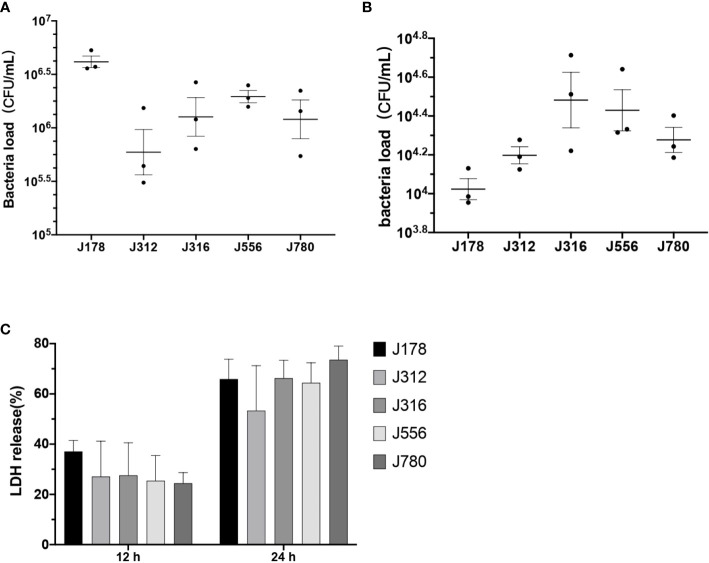
The adhesion, invasion, and cytotoxic ability of *Erwinia sorbitola* sp. *nov.* on RAW264.7 cells. **(A)** The number of bacterial loads adhered on cells; **(B)** the bacterial loads that invaded cells; **(C)** LDH released in the medium was used to assess the cytotoxicity of *Erwinia sorbitola* sp. nov. (Strains: J178, J312, J316, J556, and J780^T^. Infection dose: MOI 100. Adherence time: two hours and two hours of antibiotic treatment to kill the exocellular bacteria. All the assays were repeated three times, and each experiment was performed in triplicate. Results were presented as Mean ± SD).

As the results shown in **Figure 5**, the bacteria burdens attached to cells ranged from 10^5.4^ to 10^6.7^ CFU/mL after two hours of coculture (**Figure 5A** **)**. The bacteria burdens that had invaded cells ranged from 10^4^ to 10^4.7^ CFU/mL after two hours of inoculation and two hours of antibiotic treatment (**Figure 5B**). The cytotoxicity of *E. sorbitola* sp. nov. on cells ranged from 20-40% at 12 hours post-infection and significantly increased at 24 hours post-infection compared with 12 hours post-infection (**Figure 5C**). All these results were consistent with the predicted information. These results indicated that *E. sorbitola* sp. nov. might have pathogenicity on mice cells.

## Discussion

### Phytopathogenic mechanisms of *E. sorbitola* sp. nov.

Infection experiments on plant leaves and pear fruits in this study (**Figure 2**) identified the pathogenicity of *Erwinia sorbitola* sp. nov. but its mechanisms are still unclear. Using bioinformatics analysis with comparison to other closely related plant pathogens (**Figures 3D**, **4A–C**), we preliminarily identified and analyzed several possible points related to its pathogenicity: motility, biofilm formation, and exopolysaccharides (EPS) amylovoran; stress survival and siderophores; and Type VI secretion system (Bell et al., 2004).

Firstly, Pili synthesis was predicted in *E. sorbitola* sp. nov. A former study determined that the defect of pili resulted in decreased virulence (Koczan et al., 2011). Also, the phytopathogenic determinant amylovoran was identified in the J780^T^ genome and shared a high degree of homology with the plant pathogen *E. amylovora* (**Figure 3D**); exopolysaccharides (EPS) amylovoran also joined in biofilm formation and motility of microbial pathogens in the host plants, and the mutants deficient in amylovoran production were found to be not phytopathogenic (Bellemann et al., 1994; Kharadi et al., 2019). Flagella and pili together with amylovoran played vital roles in the survival of the bacteria outside the host and also for the attachment to host cell surfaces (van der Bosch et al., 1980; Connell et al., 1996; Rocha et al., 2007; Santander et al., 2014), which supported the above conclusion.

Secondly, *E. sorbitola* sp. nov. may significantly enhance its adaptability to adverse environments, primarily through its catalase and metal ion transport systems (**Supplementary Table 5**). *katA* and *katG* were found to contribute to fire blight symptoms and the progression of necrosis in immature fruits (Santander et al., 2018). Moreover, it was found in this study that siderophores in both isolate genomes shared high homology to that of *D. dadantii*. It was reported that the sidtheerphore in *D. dadantii* (previously *Erwinia chrysanthemi*) could cause metal homeostasis disturbances and callose deposition in host plants. Also, it contributes to a bacterial antioxidant effect by reducing ROS accumulation in bacteria (Dellagi et al., 2005; Kieu et al., 2012).

Although T3SS, a critical virulence factor of species in genus *Erwinia*, was not identified in strain J316 and J780^T^, four T6SS gene clusters were found from this new species of *Erwinia* (**Supplementary Table 6**). This study showed that the T6SS-2 and auxiliary gene clusters in the type VI secretion system of *E. sorbitola* sp. nov. shared a high homology to that of *E. amylovora* (**Supplementary Table 7**). It has been reported that the T6SS-2 mutant *E. amylovora* strain had significantly reduced virulence, possibly caused by amylovoran secretion reduction (Tian et al., 2017).

Only a part of the phytopathogenic determinants was identified and characterized in this study. There must be other determinants that still need to be discovered. Further, exploring more phytopathogenic determinants will contribute to finding the pathogenic mechanisms of *E. sorbitola* sp. nov.

### Animal pathogenicity

Adherence, invasion, and cytotoxicity assays were applied in this study to confirm the capacity of this new pathogen regarding adherence, invasion, and cytotoxicity (**Figure 5**). Several determinants related to animal pathogenicity were identified and characterized in this study as well. Furthermore, the reference strains *E. persicina* (Mohamaden et al., 2019; Ceylan and Ozden, 2022)*, E. tasmaniensis* (Shin et al., 2008a) and *E. billingiae* (Prod'homme et al., 2017; Bonnet et al., 2019) were related to several human infectious diseases. This study speculated that *E. sorbitola* sp. nov. may possess specific animal pathogenicity. However, more exploration of the animal pathogenicity of *E. sorbitola* sp. nov. should be done for further analysis.

## Conclusion

In summary, this study isolated and identified a novel species (*E. sorbitola* sp. nov.) in the genus *Erwinia* from ruddy shelducks. This study characterized it as a phytopathogen through pathogenicity analysis based on genomic sequences and plant virulence assays. Moreover, a primary pathogenetic analysis based on the model of animal cells suggested it might also have a degree of animal pathogenicity.

Emerging infectious diseases are often caused by new microorganisms. Several pre-identifications of pathogens have been reported to cause human infections. Reverse microbial etiology is one way to study emerging infectious, and the methods of this study followed previous presented procedures (Xu, 2019). When emerging infectious diseases affect plants, primarily commercial crops, it can cause substantial economic losses. Our research focused on the early isolation and identification of novel pathogens to provide faster and more precise ways for the prevention and control of emerging infectious diseases, such as the new pathogen Erwinia sorbitola sp. nov. discovered in this study, might provide the early diagnosis and control for an unknown infectious disease.

## Data availability statement

The datasets presented in this study can be found in online repositories. The names of the repository/repositories and accession number(s) can be found in the article/**Supplementary Material**.

## Ethics statement

The animal study was reviewed and approved by Ethical Committee of the National Institute for Communicable Disease Control and Prevention, Chinese Center for Disease Control and Prevention (#: ICDC-2016004).

## Author contributions

JX and HZ contributed to conception and design of the study and foundation support. JY, WS, DJ organized the database, YT, HL, SL performed the statistical analysis. YT, YG, ZZ, WL wrote the first draft of the manuscript. YH wrote sections of the manuscript. All authors contributed to manuscript revision, read, and approved the submitted version.
