# Supplementary material for: A novel phytopathogen Erwinia sorbitola sp. nov., isolated from the feces of ruddy shelducks
Source: Front Cell Infect Microbiol. 2023 Feb 16;13:1109634. doi: 10.3389/fcimb.2023.1109634 (PMC9978198; doi:10.3389/fcimb.2023.1109634)
Supplement: Supplementary file 1 [file DataSheet_1.docx]

**Fig. S1. ML (a) and MP (b) trees based on partial 16S rRNA gene sequences** **showing the phylogenetic relationship between strains J780^T^ and J316, and related taxa in the genus *Erwinia*.** (Numbers on the tree indicate bootstrap values calculated for 1000 replications for branch points > 50 %).

**(A)**

***Erwinia sorbitola* J316** **(MN708966)**

***Erwinia sorbitola* J780^T^** **(MN203624)**

*Erwinia* *rhapontici* ATCC 29283^T^ (U80206)

*Erwinia aphidicola* DSM 19247^T^ (FN547376)

*Erwinia persicina* GDMCC 1.331^T^ (BCTN01000053)

*Erwinia tasmaniensis* Et1/99^T^ (AM055716)

*Erwinia piriflorinigrans* CFBP 5888^T^ (GQ405202)

*Erwinia uzenensis* YPPS 951^T^ (AB546198)

*Erwinia amylovora* NBRC 12687^T^ (BAYW01000035)

*Erwinia pyrifoliae* DSM 12163^T^ (FN392235)

*Erwinia billingiae* CIP 106121^T^ (JN175337)

*Erwinia endophytica* BSTT30^T^ (LN624761)

*Erwinia toletana* CECT 5263^T^ (FR870447)

*Erwinia iniecta* B120^T^ (JRXE01000057)

*Erwinia typographi* Y1^T^ (GU166291)

*Erwinia gerundensis* EM486^T^ (FJ611847)

*Erwinia oleae* DAPP-PG 531^T^ (GU810925)

*Erwinia mallotivora* DSM 4565^T^ (AJ233414)

*Erwinia papayae* CFBP 5189^T^ (AY131237)

*Erwinia psidii* LMG 7039^T^ (JQ809696)

*Erwinia tracheiphila* LMG 2906^T^ (Y13250)

*Erwinia teleogrylli* SCU-B244^T^ (KF500917)

*Pseudomonas aeruginosa* LMG 1242^T^ (Z76651)

99

92

91

90

61

96

85

56

83

67

51

61

63

0.02

**(B)**

***Erwinia sorbitola* J316 (MN708966)**

***Erwinia sorbitola* J780^T^ (MN203624)**

*Erwinia rhapontici* ATCC 29283^T^ (U80206)

*Erwinia aphidicola* DSM 19247^T^ (FN547376)

*Erwinia persicina* GDMCC 1.331^T^ (BCTN01000053)

*Erwinia billingiae* CIP 106121^T^ (JN175337)

*Erwinia tasmaniensis* Et1/99^T^ (AM055716)

*Erwinia piriflorinigrans* CFBP 5888^T^ (GQ405202)

*Erwinia uzenensis* YPPS 951^T^ (AB546198)

*Erwinia amylovora* NBRC 12687^T^ (BAYW01000035)

*Erwinia pyrifoliae* DSM 12163^T^ (FN392235)

*Erwinia endophytica* BSTT30^T^ (LN624761)

*Erwinia toletana* CECT 5263^T^ (FR870447)

*Erwinia gerundensis* EM486^T^ (FJ611847)

*Erwinia oleae* DAPP-PG 531^T^ (GU810925)

*Erwinia iniecta* B120^T^ (JRXE01000057)

*Erwinia typographi* Y1^T^ (GU166291)

*Erwinia mallotivora* DSM 4565^T^ (AJ233414)

*Erwinia papayae* CFBP 5189^T^ (AY131237)

*Erwinia psidii* LMG 7039^T^ (JQ809696)

*Erwinia tracheiphila* LMG 2906^T^ (Y13250)

*Erwinia teleogrylli* SCU-B244^T^ (KF500917)

*Pseudomonas aeruginosa* LMG 1242^T^ (Z76651)

82

98

100

73

82

71

87

96

88

90

68

57

53

54

**Fig. 2. The virulence test on pear fruits of *Erwini*a *sorbitola* sp. nov. and reference strains.** Virulence test of *E. sorbitola* sp. nov. by injection to pear fruits (inoculation dose 10^7^CFU/ml).

**
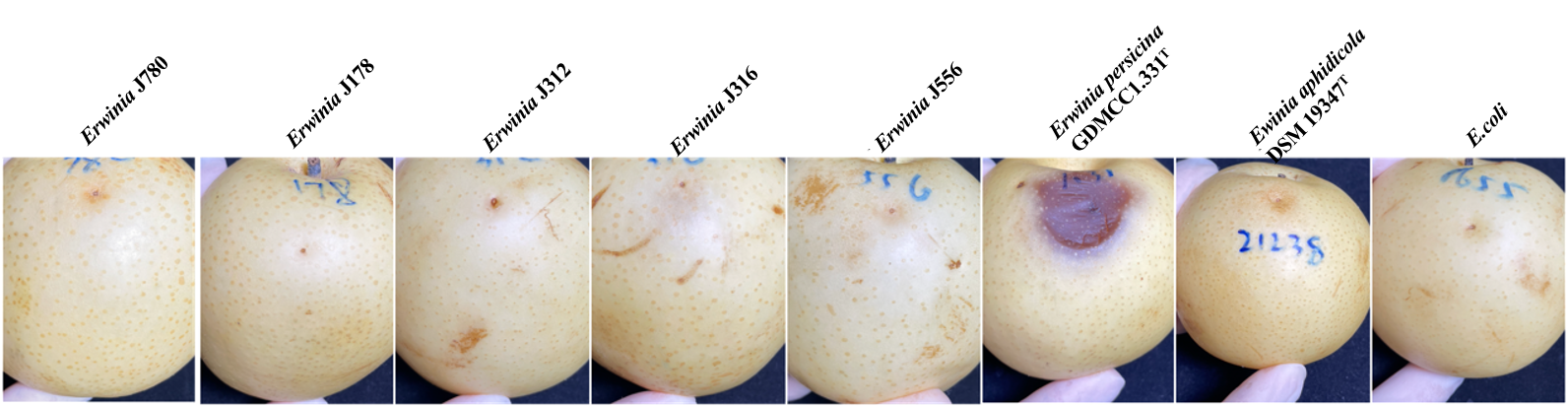
**
